# The National Eclipse Weather Experiment: use and evaluation of a citizen science tool for schools outreach

**DOI:** 10.1098/rsta.2015.0223

**Published:** 2016-09-28

**Authors:** Antonio M. Portas, Luke Barnard, Chris Scott, R. Giles Harrison

**Affiliations:** Department of Meteorology, University of Reading, Earley Gate, PO Box 243, Reading RG6 6BB, UK

**Keywords:** citizen science, schools outreach, e-science, meteorology, astrophysics, solar eclipse

## Abstract

The National Eclipse Weather Experiment (NEWEx) was a citizen science project for atmospheric data collection from the partial solar eclipse of 20 March 20. Its role as a tool for schools outreach is discussed here, in seeking to bridge the gap between self-identification with the role of a scientist and engagement with science, technology, engineering and mathematics subjects. (The science data generated have had other uses beyond this, explored elsewhere.) We describe the design of webforms for weather data collection, and the use of several external partners for the dissemination of the project nationwide. We estimate that up to 3500 pupils and teachers took part in this experiment, through the 127 schools postcodes identified in the data submission. Further analysis revealed that 43.3% of the schools were primary schools and 35.4% were secondary. In total, 96.3% of participants reported themselves as ‘captivated’ or ‘inspired’ by NEWEx. We also found that 60% of the schools that took part in the experiment lie within the highest quintiles of engagement with higher education, which emphasizes the need for the scientific community to be creative when using citizen science projects to target hard-to-reach audiences.

This article is part of the themed issue ‘Atmospheric effects of solar eclipses stimulated by the 2015 UK eclipse’.

## 1. Introduction

### (a) Background

On the morning of 20 March 2015, the Moon’s orbital path crossed in front of the Sun, casting a shadow over planet Earth. For this event, the UK was one of the few locations privileged to experience a significant partial eclipse. Eclipses have long stimulated the imagination of humankind, even though they are a well-understood consequence of Keplerian planetary motion. Although they have been thoroughly studied and their astronomical predictability has been known for centuries, questions remain over the consequences for the weather of the short-lived lunar shadow in the atmosphere. This astronomical event provided a novel opportunity to investigate some of these weather-related questions. Through involving schools across the UK to provide a huge source of observant young citizen scientists, we recognized that addressing the scientific questions could be combined with a strongly purposed science outreach activity. This combination of motivations provided the impetus for what became known as the National Eclipse Weather Experiment (NEWEx).

Citizen science (CS) projects continue to bloom across the academic community, and a considerable body of literature now exists that focuses not only on the science outcomes but also on the experiences of the participating volunteers and scientists. Unarguably, the exponential growth of modern technologies has catalysed successful CS projects, such as those forming part of the Zooniverse (www.zooniverse.org) (e.g. Solar Stormwatch, www.solarstormwatch.com [1]) or Open Air Laboratories (www.opalexplorenature.org). These allow the use of websites or apps for data collection or analysis of large datasets, avoiding a specialized level of scientific knowledge yet still bringing tangible positive outcomes to those who get involved. For instance, the ISPEX project [2] developed and distributed a low-cost add-on for smartphones that converted them into spectropolarimetric instruments to obtain high-resolution maps of aerosol properties. Through a nationwide campaign on 8 July 2013, this resulted in roughly 6000 measurements of the concentration of aerosols in the atmosphere, when the weather forecast was favourable for the experiment.

A central question remains as to what motivates volunteers, especially students and teachers, to take part in CS projects. A study conducted by Raddick *et al*. [3], under the Galaxy Zoo project, points to ‘contribution’ to a original scientific research as a major motivation to engage with this particular CS project to classify the morphology of galaxies obtained from large datasets. According to these authors, different citizen scientists will be driven by their own set of individual motivations, and more research is needed to shed light on the science learning outcomes, if any, gained by the contributing volunteers.

On the other hand, a study conducted among the astronomy scientific community gives us some insights into scientists’ views on education and public outreach [4]. The authors of that study highlighted the fact that astronomers develop their scientific aptitude from a very early stage (often at primary school level), and therefore they see the benefits of exposing youngsters to science, technology, engineering and mathematics (STEM) related activities. The authors also point out that outreach is still seen by many peers as a hobby and it often lacks financial support from grants and policy-makers, alongside a lack of encouragement from senior staff within academic departments.

Earlier familiarization with STEM subjects was also the topic of a recent report commissioned by the Women In Science and Engineering (WISE) organization under their campaign ‘People like me’ [5] (www.wisecampaign.org.uk/uploads/wise/files/not_for_people_like_me.pdf). The author explores reasons for the UK shortage of skilled workers in STEM, which reinforced the idea that more needs to be done to engage with schools-based audiences. This is especially so for those from under-represented backgrounds in higher education (HE) institutions.

Changes in the HE landscape in England and Wales during the last decade translated into universities cementing their commitment to engage positively with their local communities, schools and colleges through outreach programmes, and, specifically, boosting the widening participation agenda (see [6] for a chronological summary). These programmes often extend down from the university at institutional level to individual departments. To do so, they rely heavily on the altruistic dedication of academics and postgraduate students, who see outreach and public engagement with science as a vehicle for dissemination of their own research, as well as a gateway to improve their communication skills.

In contextualizing NEWEx within this ecosystem, we believe that this CS project encouraged those who took part to self-identify with the role of a scientist, as it was clearly capable of drawing in a wide profile of schools nationally, which would provide feedback about data collection, science outcomes and the legacy of the project.

### (b) Aims of the paper

An assessment of the performance of the CS weather observations collected by volunteers for NEWEx is discussed by Barnard *et al*. [7] in this issue. In this paper, we focus on its use as a tool for schools outreach and reflect on the design of the data collection activity through the use of webforms. We consider particularly the outreach side of the project, especially where relevant for schools and colleges across the UK. We also discuss the dissemination of the experiment achieved by working alongside national platforms such as the BBC’s *School Report*, give an analysis of who took part in the experiment, and finish with a summary of recommendations potentially useful in undertaking future events of a similar nature. Section 2 is dedicated to the description of the design and implementation of data collection from the perspective of usability. In §3, we draw a profile of the schools that collected data during the experiment, and in §4, we explore the post-experiment feedback from users who had taken part in the experiment. Finally, in §5, we draw our conclusions, not only establishing a set of recommendations as to how similar weather data collection activities might be implemented for future eclipses, but also considering the impact of similar CS projects on school audiences.

## 2. Data collection for the National Eclipse Weather Experiment

### (a) Science considerations

The major science motivation for this project was to investigate the effects of a substantial solar eclipse on the weather. This science activity effectively began with Clayton [8], who brought together weather measurements over a wide area during the US solar eclipse of 1901. Further advances were made from the dual-site work of Aplin & Harrison [9] made in cloudy and clear weather during the total solar eclipse of 11 August 1999. This study used high temporal resolution meteorological data to observe the short-term eclipse-related changes in temperature, wind speed, wind direction and cloud breakage from two meteorological sites in the UK, the Kehelland Met Office near Camborne, Cornwall, and the University of Reading Atmospheric Observatory. The authors found an eclipse-induced decrease in surface air temperature of up to 3°C in the clear conditions at Reading, and less than 1°C in the cloudy conditions in Cornwall. The effects on wind speed, wind direction and cloud breakage were rather less conclusive, although widespread public interest in the weather-related aspects of the topic was noted (www.newscientist.com/article/mg17623712-100-moons-shadow-stirs-up-eclipse-wind).

Further analysis of the 1999 eclipse using a high-resolution weather forecasting model [10] indicated the need for a denser network of observations to advance understanding of wind speed and cloud effects. This led to the idea of using a CS approach to obtain meteorological parameters across the UK during the solar eclipse of 20 March 2015—NEWEx. In addition to the science motivation of acquiring a spatially widespread and dense dataset, NEWEx presented a unique opportunity to obtain measurements from a national CS project and the strong associated science outreach possibilities.

The central aim behind NEWEx, as a CS experiment, was to encourage regular observation of simple meteorological parameters, using a webform for efficient reporting, which also allowed rapid analysis. Volunteers who took part in NEWEx were asked to collect information associated with variables similar to those previously explored by Aplin & Harrison [9]: temperature, wind speed, wind direction and cloud coverage. One of the main concerns was how to facilitate the simple measurement of these variables in order for the data to be of research use, while finding the best possible platform to collect these data on a national scale. These two factors had to be considered in the context of wide variability in the capabilities expected of the CS data collectors.

Another consideration, which is more fully explored by Barnard *et al*. [7], was how to produce rapid and tangible outcomes from the data collected, to capitalize on the interest of schools and the general public. This was also expected to infuse the data collectors with a sense of ownership and positive contribution towards the project. This latter aspect was addressed by embedding analysis infrastructure from the outset, to provide plots and updated data on regular intervals of time on part of the University of Reading Department of Meteorology’s website. On the eclipse day, these plots made the front page of national news media outlets within hours of their release.

### (b) Designing the website for data collection

The transient nature of the eclipse and the need to collect data across the entire UK argued strongly for the use of a webform, to provide a systematic, uniform and efficient collection tool. In the analysis of the total solar eclipse of 1999 [10], data values were used from the UK meteorological measurement network, which then amounted to 121 sites reporting hourly. Not all of these sites reported their data in a consistent way, which limited the analysis undertaken. Use of the webform permitted a greater density of rapid measurements. The advantages and disadvantages of collecting online data for research have been widely discussed previously [11].

The options for designing webforms lie across a spectrum, from an entirely custom design supported by a programmer, to the use of a standard tool supported on a wide variety of platforms by a major corporation. Because of the platform compatibility issue, and indeed cost, we decided to use Google Forms (www.google.co.uk/forms) to design our system, as this platform provided the necessary Web infrastructure required, and ultimately the capacity for an unknown number of users, which could possibly be very large. Google Forms also had an adequate level of adaptability needed for the basic data collection. For example, it allowed for different types of responses, such as regular text, multiple-choice entries and, among others, the ability to select an item from a list. The information required from the user was location, surface air temperature, cloud cover, wind force and wind direction, with the meteorological observations recorded at regular intervals.

Location was entered via a postcode, as the exact measurement location was not needed, and provided some level of confidentiality. (The spatial aspects of the data are explored further by Barnard *et al*. [7].) The temperature information was expected to be obtained from a simple (liquid-in-glass or digital) thermometer, typically able to be read to a resolution of between 0.1°C and 0.5°C. The wind strength was to be categorized by Beaufort force, for which the standard descriptions were given, and the wind direction by a pull-down menu of eight compass directions. Cloud amount information was simplified into three categories of overcast, broken cloud and clear. In this way, the only instrumentation actually needed was a thermometer to measure the air temperature. Basic instructions on measuring air temperature in the shade were given, following the usual meteorological practice [12]. Table 1 summarizes the range and step size of each parameter recorded by the survey.

**Table 1.** Data entries sought on NEWEx webform for data collection. The parameters used are presented, as well as the range of values employed and, when applicable, their step size.

parameter	range of values	step size
time	0800–0900; 1000–1100	15 min
time	0900–1000	5 min
temperature	−10 to 20°C	0.5°C
wind speed	Beaufort Force 0–6	1
wind direction	N–NE–E–SE–S–SW–W–NW	n.a.
cloudiness	clear sky, some cloud, much cloud, overcast	n.a.

The webforms matured through two major iterations. At each stage, concerns existed over how to balance the mixed abilities of the users with the accuracy required from the data for scientific purposes. In the first iteration of the webform, generated about a month before the eclipse, the user was asked to enter the time of observation to a resolution of 1 min and then to fill in the temperature in degrees Celsius, rounded to the nearest 0.5°C, wind speed in miles per hour and wind direction, the latter chosen from a drop-down list of compass points. This first version was released internally among peers. Feedback made it very clear that, for more than one observation, the webform would have to be repopulated each time. Clearly, this presented a substantial risk that the users would just give up on the data entry, so an improvement was sought.

**Figure 1.**
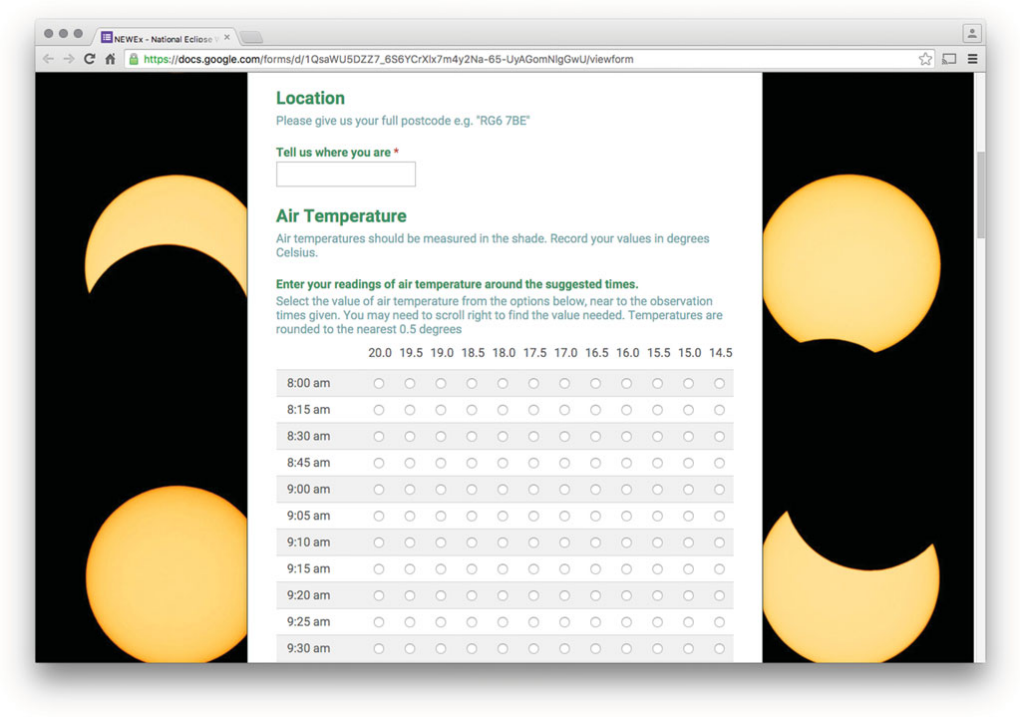
Second and final version of the webform released to the public to allow users to record their entire observations with a single submission. (Online version in colour.)

Major changes were made and a second iteration of the webform was generated, designed to avoid burdening the citizen scientists with multiple webform submissions. This revised version, as shown in figure 1, was structured so that all the observations from one observer, for the entire eclipse, could be submitted in a single submission. To do this, the observations of each meteorological variable were discretized into a matrix of radio buttons, with the columns giving the meteorological parameter values, and the rows corresponding to different observation times. This meant that numerical values were not entered individually, but instead selected from available options. This had the additional benefit of preventing entries from being mis-keyed, reducing the error checking requirement and the associated loss of data. A complete transcript of the webform is available in the electronic supplementary material.

The Google Form was embedded in the University of Reading Department of Meteorology’s website. This increased traffic to the website and, with it, the profile of the institution. However, there was a fear that if large numbers of volunteers participated in submitting data via the webform (and there was no basis for estimating the take-up in advance), both the webform and the software produced to gather and analyse the data might fail. Therefore, it was important that the complete data submission and analysis system was tested beforehand with a large number of submissions. This was done by generating synthetic meteorological observations by 10 000 observers, which were then submitted via the webform automatically, over a few minutes, as further explained in [7]. This volume of data was significantly more than that expected to be received during the eclipse, and it was demonstrated that there was no difficulty in the complete NEWEx submission and analysis system processing these.

After putting up the webpages of information about the forthcoming NEWEx activity, we handled the many ad hoc questions of how to enter data and what instruments were needed to acquire data, by producing a list of frequently asked questions on the same website. This provided a means to manage the enquiries effectively, particularly those around the type of instruments needed. Furthermore, the material was used to draw attention to important information about safe ways to observe a solar eclipse.

### (c) Dissemination methods adopted

We marketed NEWEx through a multitude of communication vehicles, with the intention of national coverage, so as to get good spatial information on the transient phenomena expected in the atmosphere. To do this, we relied heavily on third parties, such as learned societies with distribution lists, partnerships with other institutions and direct engagement with the media.

We also drew attention to the eclipse by publicizing a special in-house event. This was in the evening prior to the partial eclipse, for which we organized a series of lectures attended by 215 members of our local community. This brought together specialists not only to talk about the science but also to explore the folklore behind eclipses. This interdisciplinary approach brought together the science, history and arts associated with eclipses. Further details on the depiction of eclipses in art can be found in Blatchford in this issue [13].

For the eclipse morning itself, we sent out invitations to local schools to attend a solar eclipse event held on the Whiteknights campus of the University of Reading co-hosted by the Reading Astronomical Society (www.readingastro.org.uk), which provided a variety of telescopes and filters. Fifty-five students from both primary and secondary local schools attended this event alongside the majority of members of staff and postgraduate students of the Department of Meteorology.

At the regional level, we made use of our partnerships with the South East Physics Network, the Institute of Physics and the Royal Meteorological Society, all of whom willingly disseminated the experiment among their contacts.

A particularly fruitful collaboration arose from engagement with BBC Education, through their *School Report* (www.bbc.co.uk/schoolreport) project. This is a news report filmed and directed by school children for school children, in the past working with over 1000 schools in developing journalistic skills. This synergistic collaboration resulted in a special edition of *School Report*, with some of this paper’s authors being interviewed by local secondary school pupils (www.bbc.co.uk/schoolreport/31591588); the relationship that developed also led to contributions to the BBC *Stargazing Live* event held at Leicester Racecourse on the eclipse day, in which live regular updates from NEWEx were provided.

Even though only 26% of the participants found out about NEWEx through media coverage (see §4 for further details), we reinforce the importance of the media as a vehicle for dissemination: from local radio interviews to influential national newspapers (www.theguardian.com/science/solar-eclipse, www.dailymail.co.uk/sciencetech/article-3003941/Did-feel-eerie-wind-solar-eclipse-Onlookers-report-wind-dropping-birds-going-silent.html) and television, the unexpected ability of NEWEx to raise media interest highlighted the broad interest in the topic.

## 3. Analysis of the data

### (a) Summary of National Eclipse Weather Experiment data collection

Data submission for NEWEx began on the morning of the eclipse day (20 March 2015, a Friday) at around 7.30 GMT, with the last data entry a few days after the experiment on 27 March 2015. During this period, we received 503 webform submissions, 475 of which were submitted on the day of the eclipse, with the remainder submitted subsequently. The geographical location of each submission was determined from the participant’s postcode, which was converted by software to geographical longitude and latitude. Some multiple data entries were associated with the same postcode. We now focus on entries with postcodes matching school locations across the UK.

### (b) Data selection and validation

During the first inspection of the 503 NEWEx data entries, incomplete and duplicate postcodes were sought and removed. To study the schools’ profiles, our approach was to keep the first entry per site and discard any subsequent duplicate entries with the same complete postcode. This reduced the sample to 271 individual full postcodes. (The accompanying publication by Barnard *et al*. [7], which assessed the performance of data collected by CS volunteers, presents greater numbers, as full postcodes were not needed to validate their data entries.) We discarded partial postcodes, because we could not match them with schools or non-schools postcodes as we categorize data for further analysis.

**Figure 2.**
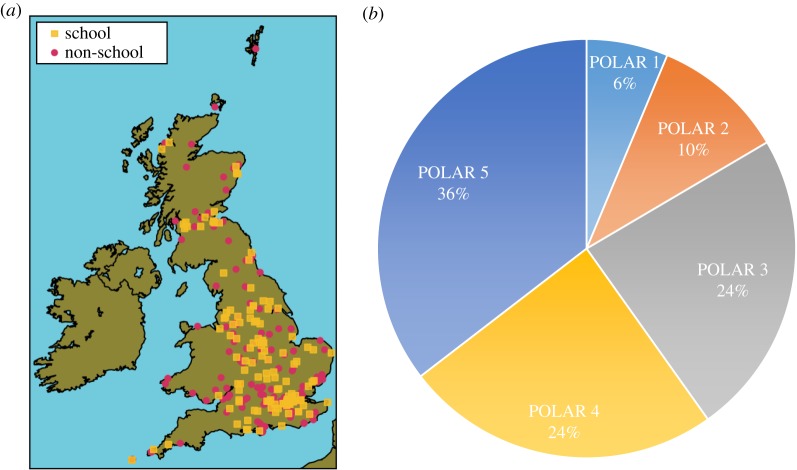
(*a*) Geographical distribution of postcodes associated with NEWEx data submission online for both schools postcodes (yellow squares) and non-schools postcodes (pink dots). (*b*) Distribution of postcodes associated with NEWEx data submission according to their quintiles of young participation with HE, also known as POLAR classification. (POLAR quintile 5 relates to the highest engagement postcodes, quintile 1 to the lowest.)

The geographical distribution of the individual postcodes can be found in figure 2*a*, where they are divided into two groups, schools and non-schools, which is a distinction developed further in this paper. In table 2, we subdivided postcodes by region to highlight the fact that the schools that participated were not evenly distributed across the country. We found a higher concentration of schools postcodes from the South East (68 out of 127), followed by Scotland (39 out of 127). By contrast, we found fewer schools postcodes from Northern Ireland (2 out of 127) and Wales (6 out of 127).

**Table 2.** Distribution of complete postcodes by region, submitted during the NEWEx. Postcodes have been categorized as originating from schools or non-schools (as explored in §3).

region	schools	non-schools	both
South East	28 (22.0%)	40 (27.8%)	68 (25.1%)
Scotland	17 (13.4%)	22 (15.3%)	39 (14.4%)
London	14 (11.0%)	4 (2.8%)	18 (6.6%)
East Midlands	13 (10.2%)	10 (6.9%)	23 (8.5%)
Yorkshire and The Humber	12 (9.4%)	5 (3.5%)	17 (6.3%)
East of England	12 (9.4%)	15 (10.4%)	27 (10%)
South West	12 (9.4%)	15 (10.4%)	27 (10%)
North West	10 (7.9%)	8 (5.6%)	18 (6.6%)
West Midlands	6 (4.7%)	11 (7.6%)	17 (6.3%)
North East	2 (1.6%)	6 (4.2%)	8 (3%)
Wales	1 (0.8%)	6 (4.2%)	7 (2.6%)
Northern Ireland	0 (0.0%)	2 (1.4%)	2 (0.7%)
total	127 (100%)	144 (100%)	271 (100%)

### (c) Demographics of contributing schools

Postcodes from entries were checked with the Department for Education Edubase2 website (www.education.gov.uk/edubase/home), which holds information on educational establishments in England and Wales. When postcodes were found to be Scottish, we made use of the Scottish, Government equivalent online database (www.educationscotland.gov.uk/parentzone/myschool/findaschool/). We found that 127 (47.2%) of the postcodes submitted were associated with schools against 144 (52.8%) of the postcodes belonging to non-schools. Out of the 127 school postcodes, 55 belonged to schools identified as Primary (43.3%), and 45 to schools identified as Secondary (35.4%); for a further 27 establishments this distinction was not applicable (21.3%).

Postcodes associated with schools were then analysed further, motivated by the widening participation agenda mentioned in the introduction of this paper. As mentioned above, HE institutions aim to become more diverse by seeking students from under-represented backgrounds. In the UK, every postcode is associated with a measurement of participation rates in HE (www.hefce.ac.uk/pubs/year/2012/201226/), also known as Participation of Local Areas (POLAR) classification. This database is maintained by the Higher Education Funding Council for England (HEFCE) and each individual postcode falls within one of five quintiles identified by POLAR. Areas with the least young participation rates fall within quintile 1 (POLAR 1) and those with the greatest young participation rates fall within quintile 5.

Cross-referencing of the NEWEx postcodes with the POLAR data classification is presented in figure 2*b*. This shows that the engagement of schools with NEWEx came predominantly from those in the quintiles 3 to 5, who, following the definition of this category, already have high rates of participation with HE. Quintile 4 (24%) and quintile 5 (36%) together contribute 60% of the participating postcodes.

## 4. Feedback

After the eclipse data collection itself was over, a further Google Form was devised and the link sent out to participants for feedback. (The questions asked are shown in the electronic supplementary material supporting this article.) The feedback form was divided into two sections. A first section contains general questions about the user, such as their location, category and overall experience. A further section was schools-related, as we wanted to unveil the impact and legacy of the project beyond the data collection and quasi-live results, as further explored by Barnard *et al*. [7]. The information obtained from this analysis was both quantitative and qualitative. A total of 31 responses were obtained between 27 March and 30 March. Twenty-seven respondents were identified as schools, two as general public and one as other.

### (a) Sample of survey

The 27 feedback responses that identified themselves as schools constituted 22% of the original number of NEWEx schools postcode data. Nonetheless, only 20 of these could be matched with the postcode data collected during the eclipse. This means that only 16% of the schools submitting data during the eclipse responded to our post-eclipse survey. There is no doubt that such a discrepancy could be easily attributed to human input error or inconsistency between the location where observations were taken (within the vicinity of the school) compared with the actual postcode of the school.

The much-reduced overall response suggests that the main commitment to the science activity was an important motivator in the original NEWEx participation. We can further speculate that seeking feedback nearly a week after the partial solar eclipse might also have reduced the number of returns. Nevertheless, we believe 22% to be a useful response level, and the very specific nature of the comments provided led us to consider them further.

### (b) Demographics

**Figure 3.**
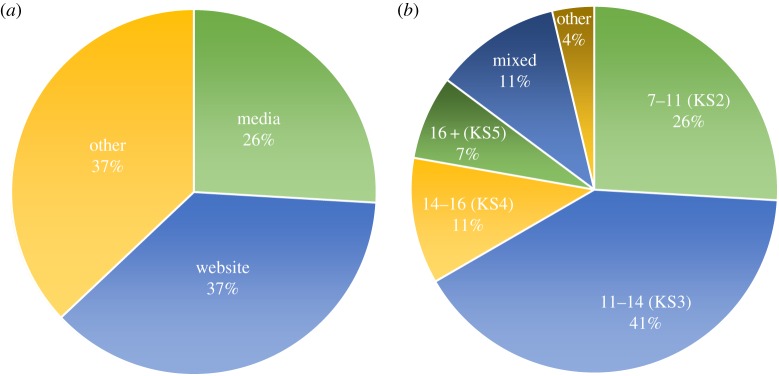
Sources of information about the project (*a*) and age groups of pupils who collected data for NEWEx (*b*) according to the feedback survey.

We found that 37% of participating schools heard about the experiment via the Department of Meteorology’s website, 26% via media coverage and the remaining 37% through other routes. As for the age groups of the pupils involved in the experiment, we found that 41% were 11–14 years old (corresponding to Key Stage 3), and 26% of pupils were between 7 and 11 (Key Stage 2). We present both quantitative results in figure 3. When asked about the impression made on pupils by the activity, 96.3% of respondents were ‘captivated’ or ‘inspired’ by the NEWEx.

It is impossible to calculate the precise number of school students involved in the experiment, because we did not explicitly ask for specific numbers of CS volunteers involved in the experiment, but ranges instead. Our most conservative estimate (based on the ranges plus exact numbers provided by some schools) indicates between 577 and 775 pupils. We can only speculate about the total number of students involved nationwide in NEWEx, as to do so requires the assumption that the 22% of schools providing feedback provide a good statistical representation of the groups of schools participating. Extrapolating on this basis in the remaining 78% of schools that did not respond to our post-eclipse survey conservatively points towards the involvement of 2600–3500 pupils in NEWEx.

The feedback survey also revealed important anecdotal feedback, which we quote below. Transcripts can be found in the electronic supplementary material to this article. Overall, pupils used their own data ‘to draw some line graphs’. One respondent pointed to the benefits of the availability of their own data: ‘We produced graphs of all the data we had collected every 2 minutes - temperature, pressure, humidity, wind speed - which allowed pupils to draw their own conclusions very quickly.’ In another context, pupils downloaded the maps released on the departmental website and explained any trends: ‘We looked at our location in particular and we tried to get the children to see if it tied-in with their raw data.’

### (c) Usability of webform as a tool to collect data

The feedback survey revealed that 92.6% of the respondents found data entry on the webform to be ‘easy’, with the remaining 7.4% responding ‘other’, which we will explore next. Only one of the respondents found it ‘really poor - could not see the time slots or criteria at the top of the page when I scrolled across to enter data?…’, suggesting that a freeze frame could be helpful to guide the user through the input table.

Another respondent commented on data entry being easy, nonetheless ‘…we had extra info to do with pressure/humidity/data every 2 minutes so an “attach” option would have been good’. Indeed, some schools subsequently sent in their data by email to the Department of Meteorology directly in case it could be used for further analysis. In both cases, the nature of the comments reflects the limitations of the generic webform platforms rather than the way the data collection was designed. As mentioned in §2, with the second iteration of the webforms, we focused on fixing and sampling time intervals around the peak of the eclipse in order to streamline data entry, so we regard that process (of initial trial and revision) as having been rather successful.

### (d) Participation aspects of the experiment

Participants were also invited to provide feedback on participating in the experiment. Overall we found positive feedback on the engagement with the CS project.

‘We enjoyed being part of this - even if the skies were full of cloud!’, reports back one of the participants. In fact, local weather may have dictated the engagement with the project because, when skies were clear, the astronomical phenomena became the principal interest: ‘We did end up giving up on the experiment - but only because the pupils being 10 years old struggled with the excitement of the eclipse and taking readings at the same time! This is through no fault of the way the experiment had been set up by yourselves. Everything was fully explained and clear to follow.’ Obviously, for many of the participants, this was probably the first eclipse they had experienced, and this would take precedence over the weather experiment. Nevertheless, if the eclipse was not visible at a site due to cloud, the eclipse occurrence could, at some sites, only have been evident from the temperature measurements.

Interestingly, Barnard *et al*. [7] report that CS volunteers collected half of the data during the peak of the eclipse and the other half prior to and after the peak, which could be interpreted as symptomatic of a genuine interest in the science goals associated with NEWEx. Unfortunately, we cannot further speculate about the volume and frequency of data submitted against cloudiness *in situ*, which could shed some light on the most prevalent motivations of CS volunteers in terms of direct participation in science versus observation of natural phenomena.

Revisiting the work of the Zooniverse paper [3] around motivations of volunteers who took part in Galaxy Zoo, ‘contribution’ towards original scientific research gathers 40% of responses. It was never a primary goal of our project to conduct such a thorough investigation on motivations behind participating in NEWEx. The study conducted in [3] made use of a robust Likert scale applied to a matrix of motivations; volunteers were asked not only to rank those, but also to choose the most important motivation. Our approach was qualitative, allowing respondents to provide additional comments on their involvement with NEWEx. Raddick *et al.* [3] emphasize the need to compare the motivations behind their Galaxy Zoo project with those of other CS projects. This could be seen as a challenge for future NEWEx- like experiments.

We also revisit the idea mentioned in §1 on the need for the scientific community to expose school audiences to positive STEM-related activities from an early age to create a long-lasting impact and combat the shortage of STEM graduates in the UK. This was supported by some of the following comments: ‘Opening up the investigation to the public allowed children in our school to work on a real life situation, using equipment they were unfamiliar with, working as a team and interpreting data.’ One of the participants added that ‘…the experiment was a really good way of getting students excited about science and because it was real data collection for a real project, it gave them a taste of what being a scientist is really like’.

Even though NEWEx facilitated a short-lived window of useful skills that scientists employ to conduct research, more needs to be done by practitioners to combat stereotypes associated with scientists and science, as discussed by MacDonald [5]. As the author further explains, in order to bridge the gap with an STEM identity, younger audiences need to be exposed to 10 different types of scientists (profiled in this study), all with unique skillsets beyond their academic knowledge. We like to think that NEWEx allowed volunteers to identify with the ‘investigator’, ‘explorer’ and ‘service provider’ profiles and that the work of MacDonald [5] will be of value when designing future CS projects, as it can help to engage with hard-to-reach audiences.

How to include hard-to-reach audiences, such as POLAR schools belonging to quintiles 1 and 2 (both demographics add up to 16% of the overall schools who engaged with NEWEx), is a current and complex question among practitioners. Producing a rigid set of guidelines of proven effectiveness seems unachievable, as there are so many unforeseen and unexplored factors in nature versus nurture (e.g. parental engagement, school infrastructure, quality of teaching). In addition, an open question remains of how to deal with audiences that simply do not want to engage with STEM-related activities.

Finally, we would like to point the reader to one apposite comment which summarizes the goals we set ourselves when we first devised NEWEx as a CS tool for schools outreach: ‘Our entire School participated, as we have a total of 38 children from age 5–11. Each child pretended to be scientists, astronomers and meteorologists! They actually were indeed “citizen scientists”, thanks to your outreach to the whole of the UK. This participation experience combined with the actual eclipse viewing really touched each of our students. We cannot thank you and the BBC enough for this incredible opportunity to learn, have fun, and see how wonderful it can be to connect with nature and science. We hope our session with the kids watching the eclipse and actually collecting data for your project, has planted at least one seed in the mind of a child that will inspire the next generation of eminent scientists in the UK! A heartfelt thanks to you all, and with our kindest regards.’

## 5. Summary and recommendations

In this paper, we have presented NEWEx as a CS tool for schools outreach, covering the issues of designing, facilitating and disseminating the collection of data through the use of online surveys or webforms. We have also explored the demographics of schools taking part in the CS project, together with their feedback about the experience. We summarize the highlights of our findings below:

— A fundamental part of NEWEx was conveying the enthusiasm for the science opportunity in getting schools across the UK involved in weather data collection for the 20 March 2015 solar eclipse; Barnard *et al.* [7] report that half of the data submitted coincide with the peak of the eclipse, which can be seen as a genuine interest from volunteers to contribute to scientific research.
— Postcode analyses mapped 47% of NEWEx submissions to schools.
— Schools that participated were not evenly distributed across the country, with a high proportion (22%) coming from the South East. In total, 43.3% of schools were identified as primary, 35.4% as secondary and for the remaining 21.3% of schools this distinction was not applicable.
— Some 60% of schools that took part in NEWEx were found to be situated in postcodes where the engagement levels with HE as classified by HEFCE falls within the two highest quintiles denoted POLAR 4 and 5.
— Post-experiment feedback data indicated that 2600–3500 pupils and their teachers took part in this CS project. In total, 96.3% of the participants responding were ‘captivated’ or ‘inspired’ by NEWEX.
— Anecdotal feedback shows a good level of satisfaction with the usage of the webform for data submission and tangible educational outcomes of the project, as teachers made the best usage of the data in their classroom.

The following points should be seen as general recommendations for future NEWEx-like CS projects:

— Webforms were found to be a reliable tool to use for national data collection. Careful webform design needs to take into consideration the wide abilities of the data collectors. Simplifying the data collection (e.g. fixing the times of the observations and minimizing the number of instruments necessary) were strong themes in our webform design.
— Webforms associated with data gathering, should include a simple identification of the data collector (e.g. school, public, other).
— Use of partner organizations was of extreme importance in engaging with a nationwide audience. The combination of local partners and national partners (particularly BBC Education’s *School Report*) catapulted NEWEx into the media and into schools.
— In addition to questions that help draw the demographics of participating volunteers, feedback forms could include an in-depth motivation matrix survey to thoroughly understand what motivates volunteers to participate in CS projects.

The popularity of CS projects has been increasing over the last decade and the evidence drawn from NEWEx here shows that they can play an important role in positively engaging students with science. We hope that our findings will be of great help not only to improve future NEWEx-like activities, but also to general outreach and public engagement practitioners.

## Supplementary Material

NEWEx data submission and feedback webforms

## Supplementary Material

NEWEx - postcodes and feedback data

## Data accessibility

The datasets supporting this article have been uploaded as part of the electronic supplementary material.
