# Supplementary material for: The National Eclipse Weather Experiment: use and evaluation of a citizen science tool for schools outreach
Source: Philos Trans A Math Phys Eng Sci. 2016 Sep 28;374(2077):20150223. doi: 10.1098/rsta.2015.0223 (PMC5004053; doi:10.1098/rsta.2015.0223)
Supplement: NEWEx data submission and feedback webforms [file rsta20150223supp1.pdf]

Edit this form

NEWEx - National Eclipse Weather Experiment

The Department of Meteorology of the University of Reading invites you to take part in a citizen science project to collect weather data during the solar eclipse on the morning of the 20th of March 2015.

To do this we would like you to make measurements of temperature, cloud and wind, and report your observations here!

We are providing you with suggested times for the observations as well as a range of values of temperatures, clouds and wind force. It is OK to take your observations a few minutes before or after the suggested times! If you can't make measurements at all the times, please still enter the ones that you have.

The uploaded data will be used to study temperature, wind and cloud changes during the eclipse. For your data to be considered for inclusion in Stargazing Live, we will need the values to be submitted by 12:00 GMT on the 20th March. The webform will, however, remain open for other submissions until 21:00 GMT in the evening.

CAUTION: Never look directly at the Sun with unprotected eyes. Look away from the Sun when estimating cloud and wind force.

* Required

Location

Please give us your full postcode e.g. "RG6 7BE"

Tell us where you are *

Air Temperature

Air temperatures should be measured in the shade. Record your values in

degrees Celsius.

Enter your readings of air temperature around the suggested times.

Select the value of air temperature from the options below, near to the observation times given. You may need to scroll right to find the value needed. Temperatures are rounded to the nearest 0.5 degrees

	20.0	19.5	19.0	18.5	18.0	17.5	17.0	16.5	16.0	15.5	15.[illegible]
8:00 am											
8:15 am											
8:30 am											
8:45 am											
9:00 am											
9:05 am											
9:10 am											
9:15 am											
9:20 am											
9:25 am											
9:30 am											
9:35 am											
9:40 am											
9:45 am											
9:50 am											
9:55 am											
10:00 am											
10:15 am											
10:30 am											
10:45 am											
11:00 am											

Clouds

How would you describe the sky around the suggested times ?

	clear sky	some cloud	much cloud	totally overcast
8:00 am	○	○	○	○
8:15 am	○	○	○	○
8:30 am	○	○	○	○
8:45 am	○	○	○	○
9:00 am	○	○	○	○
9:05 am	○	○	○	○
9:10 am	○	○	○	○
9:15 am	○	○	○	○
9:20 am	○	○	○	○
9:25 am	○	○	○	○
9:30 am	○	○	○	○
9:35 am	○	○	○	○
9:40 am	○	○	○	○
9:45 am	○	○	○	○
9:50 am	○	○	○	○
9:55 am	○	○	○	○
10:00 am	○	○	○	○
10:15 am	○	○	○	○
10:30 am	○	○	○	○
10:45 am	○	○	○	○
11:00 am	○	○	○	○

Wind force - Beaufort scale

Look around you and use the Beaufort wind force scale to describe the wind around you. Please use the following description for guidance:

0 - Calm - smoke rises vertically - less than 1 km/h;

1 - Light air - direction shown by smoke drift but not by wind vane - 1 to 5 km/h;

2 - Light breeze - wind felt on face; leaves rustle; wind vane moved by wind - 6 to 11 km/h;

3 - Gentle Breeze - Leaves and small twigs in constant motion; light flags extended - 12 to 19 km/h;

4 - Moderate Breeze - Raises dust and loose paper; small branches moved - 20 to 28 km/h;

5 - Fresh Breeze - Small trees in leaf begin to sway; crested wavelets form on inland waters - 29 to 38 km/h;

6 - Strong Breeze - Large branches in motion; whistling heard in telegraph wires; umbrellas used with difficulty - 38 to 49 km/h;

Record the strength of the wind around the suggested times

	0 - Calm	1 - Light air	2 - Light breeze	3 - Gentle breeze	4 - Moderate breeze	5 - Fresh breeze	6 - Strong breeze
8:00 am							
8:15 am							
8:30 am							
8:45 am							
9:00 am							
9:05 am							
9:10 am							
9:15 am							
9:20 am							
9:25 am							
9:30 am							
9:35 am							

9:40 am	○	○	○	○	○	○	○
9:45 am	○	○	○	○	○	○	○
9:50 am	○	○	○	○	○	○	○
9:55 am	○	○	○	○	○	○	○
10:00 am	○	○	○	○	○	○	○
10:15 am	○	○	○	○	○	○	○
10:30 am	○	○	○	○	○	○	○
10:45 am	○	○	○	○	○	○	○
11:00 am	○	○	○	○	○	○	○

Wind direction

From which direction is the wind blowing around the suggested times?

	N - north	NE - north-east	E - east	SE - south-east	S - south	SW - south-west	W- west	NW- north-west
8:00 am	○	○	○	○	○	○	○	○
8:15 am	○	○	○	○	○	○	○	○
8:30 am	○	○	○	○	○	○	○	○
8;45 am	○	○	○	○	○	○	○	○
9:00 am	○	○	○	○	○	○	○	○
9:05 am	○	○	○	○	○	○	○	○
9:10 am	○	○	○	○	○	○	○	○
9:15 am	○	○	○	○	○	○	○	○
9:20 am	○	○	○	○	○	○	○	○
9:25 am	○	○	○	○	○	○	○	○
9:30 am	○	○	○	○	○	○	○	○
9:35 am	○	○	○	○	○	○	○	○

9:40 am	○	○	○	○	○	○	○	○
9:45 am	○	○	○	○	○	○	○	○
9:50 am	○	○	○	○	○	○	○	○
9:55 am	○	○	○	○	○	○	○	○
10:00 am	○	○	○	○	○	○	○	○
10:15 am	○	○	○	○	○	○	○	○
10:30 am	○	○	○	○	○	○	○	○
10:45 am	○	○	○	○	○	○	○	○
11:00 am	○	○	○	○	○	○	○	○

Submit

Never submit passwords through Google Forms.

Powered by Google Forms

This content is neither created nor endorsed by Google.

Report Abuse - Terms of Service - Additional Terms

Edit this form

NEWEx - National Eclipse Weather Experiment - Feedback

The Department of Meteorology of the University of Reading would like to thank you for participating in the National Eclipse Weather Experiment. We have had over 500 responses nationwide and the preliminary results of the experiment can be found at http://www.met.reading.ac.uk/outreach/newex_2015/index.html.

We would really appreciate some feedback on your experience of the collection and input of data as well as some basic information on participating schools if applicable.

* Required

General information

Tell us where you are *

provide us with your full postcode

[ ]

What best describes you ?

- ○ school
- ○ general public
- ○ Other: [ ]

How did you find out about NEWEx - National Eclipse Weather Experiment?

- ○ media
- ○ website
- ○ Other: [ ]

How would you rate the experience of entering the data into the webform

- easy
- more instructions needed
- difficult
- gave up
- Other:

How useful did you find the quick results presented on the NEWEx webpage?

- easy to interpret
- basically accessible
- more explanation needed
- incomprehensible
- Other:

Please give us any further comments about this experiment

Schools specific feedback

School's name

Age group of pupils involved in obtaining the data

- 7-11
- 11-14
- 14-16
- 16+
- mixed
- Other:

How many pupils were involved in the experiment and data entry?

- 1-10
- 11-20
- 21-30
- Other:

How much of an impression did the eclipse weather make on the pupils involved in the experiment?

- captivated
- inspired
- indifferent
- bored

Have you attempted any follow up activity using the weather eclipse data with your pupils?

What is the percentage of free school meals in school ?

For us to identify outreach to broadening participation groups we would appreciate some information on FSM proportions, if any.

- 0%-10%
- 11%-20%
- 21%-30%
- 31% or above

Submit

Never submit passwords through Google Forms.

Powered by
Google Forms

This content is neither created nor endorsed by Google.

Report Abuse - Terms of Service - Additional Terms
